# A Systematic Review of Stem Cell Applications in Maxillofacial Regeneration

**DOI:** 10.3390/dj12100315

**Published:** 2024-09-29

**Authors:** Man Hung, Mahsa Sadri, Melanie Katz, Connor Schwartz, Amir Mohajeri

**Affiliations:** 1 College of Dental Medicine, Roseman University of Health Sciences, South Jordan, UT 84095, USA; 2 Division of Public Health, University of Utah, Salt Lake City, UT 84108, USA; 3 Department of Orthopaedics, University of Utah, Salt Lake City, UT 84108, USA; 4 Huntsman Cancer Institute, Salt Lake City, UT 84112, USA; 5 The Wharton School, University of Pennsylvania, Philadelphia, PA 19104, USA; 6 Library, Roseman University of Health Sciences, South Jordan, UT 84095, USA

**Keywords:** stem cells, bone regeneration, literature review, maxillofacial, tissue regeneration

## Abstract

Introduction: Regenerative medicine is revolutionizing oral and maxillofacial surgeries with stem cells, particularly mesenchymal stem cells, for tissue and bone regeneration. Despite promising *in-vitro* results, human trials are limited. A systematic review is needed to evaluate stem cell efficacy in maxillofacial issues, aiming to improve surgical outcomes and patient satisfaction. Methods: Following Preferred Reporting Items for Systematic Reviews and Meta-Analyses Guidelines, this review included peer-reviewed articles (2013–2023) on stem cells in oral surgery, excluding non-English publications, abstracts, reviews, and opinion pieces. Searches were conducted in PubMed, Web of Science, OVID, Cochrane, Dentistry & Oral Sciences Source—Ebscohost, and Scopus. Two authors independently screened titles and abstracts, resolving disagreements by consensus. Full-text analysis involved extracting key data, verified by a secondary reviewer and additional quality checks. Results: From 3540 initial articles, 2528 were screened after removing duplicates, and 7 met the inclusion criteria after excluding irrelevant studies. Key themes included the safety and efficacy of stem cell therapy, and bone regeneration and quality. Studies predominantly used mesenchymal stem cells. Findings showed positive outcomes in clinical safety and effectiveness and significant potential for bone regeneration. Conclusions: This systematic review highlights the potential of stem cell therapies in maxillofacial applications, supporting their safety, efficacy, and bone regeneration capabilities. Further research is needed to standardize protocols and confirm long-term benefits.

## 1. Introduction

The field of regenerative medicine has undergone a transformative shift in the therapeutic approaches employed in oral and maxillofacial surgeries, exemplifying the potential of stem cells in the regeneration of tissues and bones. Regenerative medicine, as an interdisciplinary domain, seeks to restore damaged tissues and organs by integrating principles from both life sciences and engineering [1]. Stem cells, characterized by their self-renewal and differentiation capabilities, have emerged as promising candidates to address challenges within oral surgery, particularly in the context of tissue and bone renewal [2].

Undifferentiated in nature, stem cells possess the ability to differentiate into various lineages under specific conditions [3]. Depending on their origin, stem cells may be totipotent, pluripotent, or multipotent. Totipotent cells can form entire organisms, while pluripotent cells give rise to mesodermal, endodermal, or ectodermal layers. In contrast, multipotent cells are constrained to specific subtypes of cell lineages [4]. Mesenchymal stem cells (MSCs), with their unique ability to differentiate and proliferate, play a pivotal role in tissue engineering and are found in nearly all multicellular organisms [3,5].

This systematic review aimed to address specific gaps in the literature regarding the efficacy and safety of stem cell therapies in maxillofacial regeneration. The primary objectives are to (1) evaluate the current evidence on the use of stem cells in bone and tissue regeneration specifically within human maxillofacial applications, (2) identify the types of stem cells and methodologies employed, (3) assess the outcomes and limitations of these studies, and (4) highlight areas requiring further research. By systematically reviewing these aspects, the review sought to provide clarity on the therapeutic potential and challenges of integrating stem cell applications into maxillofacial surgery.

Recent advancements in stem cell research have significantly impacted their application in maxillofacial surgeries. For example, the development of induced pluripotent stem cells (iPSCs) offers a potentially limitless source of cells that can differentiate into various tissue types required for maxillofacial regeneration [6,7]. Furthermore, the refinement of scaffold-based delivery methods has enhanced the integration and function of transplanted cells, improving the success rates of these therapies [8]. New materials like bioactive glass and 3D-printed scaffolds are being utilized to provide a more conducive environment for stem cell growth and differentiation, directly impacting their effectiveness in bone and tissue regeneration [9]. These advancements underscore the evolving landscape of stem cell applications in maxillofacial surgery, highlighting the need for continuous updates and assessments of their efficacy and safety.

*In-vitro* experiments are conducted to guide and regulate the differentiation of stem cells into specific subtypes, evaluating their potential to restore structure and function [2]. This approach holds promise for replacing lost bone in maxillofacial regions resulting from congenital abnormalities, trauma, or tumor excision [10]. The therapeutic application of stem cell regeneration hinges on the successful differentiation of stem cells into target lineages. While numerous preclinical and clinical studies have explored the efficacy of stem-cell-based therapy in oral surgery using animal models, the replacement of bone for deformities remains a formidable challenge [11,12]. A preliminary study by Pedroni et al. [10] on the potential of human dental pulp stem cells demonstrated osteogenic differentiation in cell sheets derived from human third molars. However, further *in-vivo* studies on humans are warranted, as no subsequent research has been conducted using this technique.

Several research articles highlight the use of stem cells in tissue regeneration [13,14], such as one study where stem cells derived from human dental pulp were transduced with a chondrogenic gene [14]. Electrospun polymer scaffolds were employed to seed stem cells, utilizing electrospinning as a versatile scaffold fabrication method supporting both *in-vivo* and *in-vivo* cell growth [14]. Research emphasizes the potential of stem cells in bone and tissue regeneration [15], primarily based on animal studies [16] conducted in laboratory settings. To ascertain the efficacy of stem cells in humans, further research specifically involving human subjects is imperative. This systematic review specifically aimed to address this gap by focusing on human studies and evaluating the translation of preclinical findings into clinical practice. Given the existing evidence from preclinical studies and preliminary human data, there is a strong justification for conducting a scoping review to investigate the efficacy of stem cells in addressing maxillofacial issues in humans. Such a review would comprehensively analyze the available literature, identify gaps in current knowledge, and provide a synthesis of findings to guide future research and clinical applications. By elucidating the role of stem cells and their derivatives in enhancing oral surgical outcomes, this review aimed to contribute to the development of innovative therapeutic strategies that offer improved patient and procedure safety, reduced treatment duration, minimized side effects, and the capability to treat large osseous lesions that were previously challenging with alternative therapies.

## 2. Methods

This review adhered to the Preferred Reporting Items for Systematic Reviews and Meta-Analyses (PRISMA 2020) guidelines. [17]. In order to be eligible for inclusion, articles were required to specifically address stem cells in tissue and bone regeneration in oral surgery. To ensure the review’s relevance to the latest developments in stem cells in oral surgery, the inclusion criteria were refined to encompass articles published over the past decade, from 2013 to 2023. Additional criteria mandated that eligible articles be peer-reviewed and published in English, with exclusion criteria being abstract-only presentations and those lacking full-text content. To maintain a focus on original research, review articles providing summaries of existing literature were deliberately excluded. Furthermore, conference proceedings, opinion pieces, and letters to the editor were excluded as they lacked empirical evidence. A detailed outline of the inclusion and exclusion criteria guiding the article selection process is presented in Table 1.

The initial search encompassed multiple databases, including PubMed, Web of Science, OVID, Cochrane, Dentistry & Oral Sciences Source—Ebscohost, and Scopus. These databases were chosen for their comprehensive coverage of scholarly articles, clinical trials, and research studies across various disciplines. This strategic choice aimed to enrich the diversity of sources included in the review, encompassing a broad spectrum of scientific contributions. Detailed search strategies and keywords specific to each database were employed, and a comprehensive overview of these strategies is provided in Table 2.

The development of these strategies was conducted with attention to detail, ensuring their tailored nature to maximize the retrieval of relevant articles. This approach was designed to facilitate a comprehensive and exhaustive consideration of pertinent studies within the scope of stem cells in tissue and bone regeneration in oral surgery. Initially, a screening phase was carried out by two authors (M.K. and M.S.), who independently assessed titles and abstracts based on predefined inclusion and exclusion criteria, documenting reasons for exclusion where applicable. Each author’s decisions and selected articles were then independently reviewed by one of the other authors to ensure thorough cross-checking, and any disagreements were resolved through discussion and consensus.

Articles that passed this initial screening underwent a comprehensive full-text analysis. During this phase, key information was extracted, including study design, participant demographics, and pertinent outcomes. To ensure the accuracy and completeness of the extracted data, a secondary reviewer independently repeated the extraction process. Additionally, other authors (A.M., C.S., and M.H.) conducted a thorough examination of the quality, origins, and data representation, enhancing the robustness of the systematic review process. This additional review helped to confirm that all relevant information was captured and that any discrepancies were identified and resolved.

Additionally, a formal assessment of the risk of bias was conducted for each included study using the Cochrane Risk of Bias Tool for randomized trials [18] and the ROBINS-I tool for non-randomized studies [19]. These tools assess potential sources of bias, such as selection bias, performance bias, detection bias, attrition bias, reporting bias, and other biases, allowing for a more comprehensive evaluation of the reliability of the findings from the reviewed articles. Each study was rated as having a low, medium, or high risk of bias, based on criteria specific to study design and methodology by one author (M.H.) and confirmed by two authors (M.K. and M.S.). The inclusion of a risk of bias assessment is critical for understanding the potential limitations of the evidence base and for providing a deeper interpretation of the results.

## 3. Results

**Figure 1:**
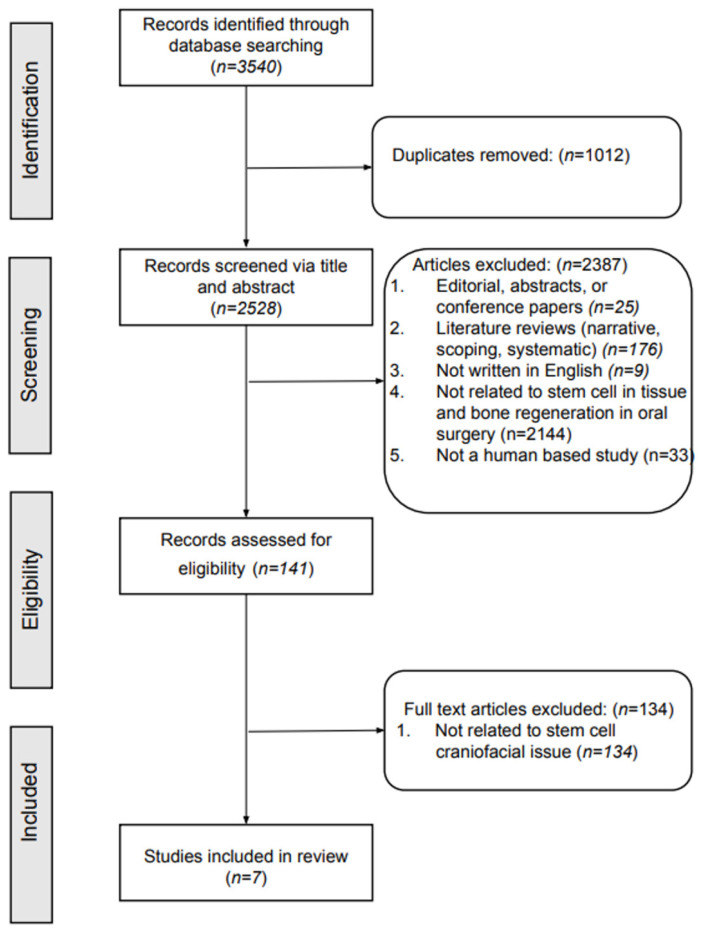
Flowchart of the article selection process.

This initial search identified 3540 articles. After removing duplicate articles, 2528 unique articles were reviewed. During screening, 2387 articles were excluded. Further refinement by applying the eligibility criteria led to the exclusion of 141 more articles that lacked relevance to stem cells and maxillofacial issues. Ultimately, 7 articles were selected for inclusion in the systematic review (Figure 1).

The findings from the review were categorized into two main themes: safety and efficacy of stem cell therapy, and bone regeneration and quality.

### 3.1. Safety and Efficacy of Stem Cell Therapy

The majority of the studies reviewed, including those by Asahina et al. [20], Feng et al. [21], Katagiri et al. [22], and Katagiri et al. [23], consistently reported that stem cell-based therapies are safe and effective in various dental surgical procedures. Across these studies, different types of stem cells, such as bone marrow stem cells, mesenchymal stem cells, and small blood stem cells, were used in procedures like sinus lifts and bone grafts. The studies found no significant side effects or health concerns, demonstrating the potential for these therapies to enhance bone regeneration and implant stability with a high degree of safety.

### 3.2. Bone Regeneration and Quality

Several studies, including those by Giuliani et al. [24], Gjerde et al. [25], and Gupta et al. [26], focused on the effectiveness of stem cells in promoting bone regeneration and improving the quality of the regenerated bone. These studies highlighted the strong osteogenic potential of stem cells, with significant new bone formation and primary stability observed in procedures like sinus augmentation and bone grafting. Moreover, the quality of the regenerated bone was noted to be structurally sound, with studies like Giuliani et al.’s [18] reporting a compact and uniformly vascularized bone structure, indicative of successful regeneration.

Table 3 provides a summary of the studies reviewed.

### 3.3. Risk of Bias Assessment

The risk of bias assessment, as detailed in Table 4, revealed varying levels of bias across the included studies. Selection bias was generally low to medium, with studies by Asahina et al. [20] and Giuliani et al. [24] demonstrating robust participant selection methods, while others by Katagiri et al. [22,23] exhibited higher selection bias due to a lack of randomization and control groups. Performance bias was universally high across studies, largely due to the difficulties in blinding participants and clinicians in surgical interventions involving stem cells. Detection bias was predominantly low in studies utilizing objective measures such as histological and radiographic evaluations (e.g., Giuliani et al. [24]), but medium in others where assessment methods were less clear. Attrition bias varied, with some studies reporting high bias due to substantial dropout rates or incomplete follow-ups, such as Gjerde et al.’s study [25]. Reporting bias was generally low to medium across studies, with transparent protocols noted in studies like Gupta et al.’s [26], but potential selective reporting was identified in others. The overall risk of bias ranged from low to high, indicating a need for cautious interpretation of the findings and highlighting areas for methodological improvement in future studies.

### 3.4. Limitations of Included Studies

Each included study had specific limitations that could impact the generalizability and reliability of the findings. For instance, the study by Asahina et al. [20] had a limited sample size and was conducted in a single center, which may not represent broader patient populations. Similarly, the study by Gjerde et al. [25] had a short follow-up period, making it challenging to assess the long-term safety and efficacy of the treatment. Moreover, studies like those by Katagiri et al. [22,23] lacked randomization and control groups, potentially introducing selection bias and limiting the robustness of their conclusions.

The methodologies used in these studies also varied significantly, which complicates direct comparisons and synthesis of the results. For example, Giuliani et al. [24] used a cohort study design with histological evaluation, whereas Gupta et al. [26] employed a case–control approach with a focus on clinical outcomes. Such methodological diversity makes it difficult to draw uniform conclusions about the efficacy of stem cell therapies. Furthermore, the geographic concentration of studies in Asia and Europe may limit the generalizability of the findings to other populations.

In light of these limitations, the findings of this systematic review should be interpreted with caution, and further research with larger, more diverse populations and standardized methodologies is necessary to validate these results.

## 4. Discussion

This systematic review provides a comprehensive examination of stem cell applications in maxillofacial therapies, revealing promising advancements while highlighting areas for further exploration. The findings align with and expand upon existing literature, offering a better understanding of the safety and efficacy of stem cell research for maxillofacial regeneration.

### 4.1. Safety and Immune Modulation

The consistent demonstration of safety in stem cell-based therapies across the reviewed studies is a crucial finding, reflecting a broader trend in regenerative medicine. As reported by Asahina et al. [20], Katagiri et al. [22,23], and others, the absence of significant adverse effects suggests that stem cell therapies are generally well tolerated in dental surgical procedures. This aligns with the extensive body of literature where stem cell-based interventions have shown a favorable safety profile in various medical applications, not just in dental surgery, but also in orthopedics and other procedures [27].

However, the immune response to these therapies remains a complex and less understood area. The elevated levels of inflammatory markers, such as eotaxin and interleukin-17A, observed by Feng et al. [21], underscore the need to better understand how implanted stem cells interact with the host’s immune system. Inflammation is a natural part of the healing process, but the balance between beneficial and harmful inflammation is delicate. Excessive or prolonged inflammatory responses could negate the positive effects of stem cell therapies, potentially leading to tissue damage, fibrosis, or even graft failure.

This finding is consistent with the broader literature, which suggests that while MSCs have immunomodulatory properties, the extent and nature of these effects can vary significantly depending on factors such as cell source, delivery method, and patient-specific variables [28]. For instance, preconditioning of MSCs with cytokines or growth factors before transplantation has been shown to enhance their immunomodulatory capacity and reduce the risk of adverse immune reactions [29]. Moreover, the immune response is not solely a concern in terms of inflammation but also in the context of stem cell survival and function. The host immune system can recognize implanted cells as foreign, leading to their rejection or impaired function, which could diminish the overall efficacy of the therapy. This is particularly relevant for allogeneic stem cell therapies, where cells are derived from donors rather than the patient. Future research should focus on understanding these immune dynamics more deeply, potentially leading to the development of more refined strategies for controlling the immune response to maximize the therapeutic benefits of stem cell interventions.

### 4.2. Efficacy and Quality of Regenerated Bone

The efficacy of stem cell therapies in promoting bone regeneration, as consistently demonstrated in the reviewed studies, is another significant finding. Not only do these therapies enhance bone formation, but they also improve the structural quality of the regenerated bone. The studies by Giuliani et al. [24] and Gjerde et al. [25] highlight that the bone produced through stem cell therapies is not just abundant but also of high quality, characterized by compactness and extensive vascularization. This is crucial for the long-term success of dental implants, as bone quality directly affects implant stability and resistance to resorption [30]. The emphasis on bone quality is supported by research suggesting that MSCs and other stem cell types have a unique ability to orchestrate the formation of bone that closely mimics native tissue. This is likely due to the paracrine effects of stem cells, where they secrete factors that modulate the local environment to favor osteogenesis and angiogenesis [31]. These paracrine effects are critical for not only the quantity of bone formed but also its integration with the surrounding tissues and its functional properties.

Moreover, the structural integrity and vascularization of the regenerated bone are vital for the success of dental implants. Vascularization ensures that the bone remains viable and that the implant site receives adequate blood supply, which is essential for the healing process and the long-term stability of the implant. Traditional bone grafting techniques often struggle to achieve this level of vascularization, particularly in larger or more complex defects, which can lead to graft failure or resorption over time. The ability of stem cell therapies to overcome these challenges suggests that they could offer significant advantages over conventional methods, particularly in complex cases involving large bone defects or severe atrophy. However, achieving these outcomes consistently requires careful consideration of factors such as the source of stem cells, the delivery method, and the use of adjunctive therapies to support bone regeneration.

### 4.3. Clinical Implications and Future Directions

The integration of stem cell therapies into maxillofacial procedures presents significant opportunities and challenges for clinical practice. Stem cell therapies, particularly those using MSCs and iPSCs, have demonstrated promising results in regenerating bone and tissue, making them viable options for patients with severe bone defects or conditions requiring extensive reconstructive surgery. However, the variability in patient responses to stem cell therapies requires careful consideration. Factors such as age, health status, and the specific stem cell types used can influence outcomes, necessitating personalized treatment plans and vigilant monitoring of immune responses to optimize efficacy and minimize risks.

Advanced biomaterials and scaffold technologies are crucial in enhancing the effectiveness of stem cell therapies. The use of 3D- or 4D-printed scaffolds, bioactive glass, and hydrogels mimics the natural extracellular matrix, promoting better cell adhesion, proliferation, and differentiation, which are essential for successful tissue regeneration. Innovations in scaffold design, such as the incorporation of growth factors and nanoparticles, have been shown to improve osteogenic potential and vascularization, particularly in complex or extensive bone defects. Continued research into next-generation biomaterials that are both biocompatible and bioactive is essential for maximizing the clinical success of these therapies.

Ethical and regulatory considerations are paramount when integrating stem cell therapies into clinical practice. The use of stem cells—whether autologous or allogeneic—raises ethical questions related to consent, long-term monitoring, and potential side effects. Regulatory bodies must establish clear guidelines to ensure that stem cell therapies are safe, effective, and ethically administered. Clinicians must also be prepared for legal and ethical challenges, especially when using genetically modified cells like iPSCs. Developing robust ethical frameworks and regulatory policies is essential to ensure patient safety and maintain public trust in these emerging therapies.

Long-term outcomes and patient monitoring are critical areas for future research. While current studies demonstrate short-term safety and efficacy, long-term data are needed to assess the durability of regenerated tissues and the stability of implants. Understanding how stem cells and their secretomes, such as exosomes, contribute to tissue regeneration over extended periods could offer insights into optimizing these therapies. Future studies should focus on long-term follow-ups to evaluate the sustained effectiveness and potential late-onset complications of stem cell treatments.

The successful integration of stem cell therapies into maxillofacial surgery requires a multidisciplinary approach. Collaboration among oral surgeons, bioengineers, immunologists, and regulatory experts is vital for developing comprehensive treatment protocols. Clinicians should be well versed not only in the technical application of stem cells but also in the biological mechanisms and potential complications associated with their use. Education and training programs should be expanded to include the latest advancements in stem cell research and its clinical applications, ensuring that practitioners are prepared to implement these therapies safely and effectively.

Addressing cost and accessibility is another crucial consideration for the widespread adoption of stem cell therapies. Current production and storage processes for stem cells, especially iPSCs and MSCs, are expensive and complex. Reducing these costs through technological advancements, such as automated cell culture systems and improved cryopreservation methods, is necessary to make these therapies more accessible. Additionally, strategies to ensure equitable access, particularly in low-resource settings, should be developed to avoid disparities in treatment availability.

Finally, future research should explore novel combinations of stem cells with other therapeutic modalities, such as gene therapy or advanced biomaterial scaffolds, to enhance regenerative outcomes. Investigating new delivery methods, such as localized versus systemic administration, could also provide insights into optimizing stem cell treatments. Expanding preclinical models that better mimic human conditions will be essential for translating these therapies from experimental studies to routine clinical practice.

### 4.4. Conclusions

This systematic review highlights the significant potential of stem cell-based therapies in maxillofacial applications, particularly for bone regeneration. The studies reviewed provide strong evidence of safety and efficacy, with promising results in terms of the quality of regenerated bone. However, the findings also underscore the complexity of the biological processes involved and the need for further research to optimize these therapies. As the field advances, stem cell-based approaches could become a cornerstone of dental regenerative medicine, offering new possibilities for patients with challenging conditions. Continued innovation and rigorous clinical evaluation will be essential to fully realize the potential of stem cell therapies in improving patient outcomes and advancing maxillofacial surgery.

## Figures and Tables

**Table 1:** Inclusion and exclusion criteria.

Inclusion Criteria	Exclusion Criteria
Focused on stem cells in tissue and bone regeneration in oral surgery Peer-reviewed in English Article published between 2013 and 2023 Subjects must be human	Articles that consisted of only abstracts without the full text Literature reviews (narrative, scoping, systematic, meta-analysis) Conference proceedings Letters for editors Animal studies

**Table 2:** Database search strategy.

Databases Database Link Search Date	Search Strategies	Number of Articles Found
Scopus https://www.scopus.com/home.uri (accessed on 2 February 2024)	(TITLE-ABS-KEY (“stem cell*”) AND TITLE-ABS-KEY (“bone regeneration” OR “tissue regeneration” OR “tissue engineering” OR “bone graft*”) AND TITLE-ABS-KEY (dent*)) AND PUBYEAR > 2013 AND PUBYEAR < 2023 AND (EXCLUDE (EXACTKEYWORD, “Animals”) OR EXCLUDE (EXACTKEYWORD, “Nonhuman”) OR EXCLUDE (EXACTKEYWORD, “Animal”) OR EXCLUDE (EXACTKEYWORD, “Animal Experiment”) OR EXCLUDE (EXACTKEYWORD, “Mouse”) OR EXCLUDE (EXACTKEYWORD, “Animal Tissue”) OR EXCLUDE (EXACTKEYWORD, “Animal Cell”) OR EXCLUDE (EXACTKEYWORD, “Rat”) OR EXCLUDE (EXACTKEYWORD, “Mice”) OR EXCLUDE (EXACTKEYWORD, “Animal Model”) OR EXCLUDE (EXACTKEYWORD, “Rats”))	1897
PubMed https://pubmed.ncbi.nlm.nih.gov (accessed on 2 February 2024)	((“stem cell*” [Title/Abstract]) AND (“bone regeneration” [Title/Abstract] OR “tissue regeneration” [Title/Abstract] OR “tissue engineering” [Title/Abstract] OR “bone graft*” [Title/Abstract]) AND “dent*” [Title/Abstract]) AND ((humans[Filter])	980
WoS https://access.clarivate.com/login?app=wos (accessed on 2 February 2024)	((TS = (“stem cell*”)) AND TS = (“bone regeneration” OR “tissue regeneration” OR “tissue engineering” OR “bone graft*”)) AND TS = (dent*) AND ((ALL = ((“population groups” not “animal models”))) OR ALL = (men OR women OR patient OR female OR male OR subjects OR adult)) NOT ALL = (“animal models”) AND (PY = (“2023” OR “2022” OR “2021” OR “2020” OR “2019” OR “2018” OR “2017” OR “2016” OR “2015” OR “2014” OR “2013”))	488
OVID https://ovidsp.ovid.com/ (accessed on 2 February 2024)	((stem cell*.ti,ab) AND ((bone regeneration.ti,ab) OR (tissue regeneration.ti,ab) OR (tissue engineering.ti,ab) OR (bone graft*.ti,ab)) AND (dent*.ti,ab) AND ((men) OR (women) OR (patient) OR (female) OR (male) OR (subjects) OR (adult)) NOT (animal models)) limit to yr = “2013–2023”	108
Cochrane Library https://www.cochranelibrary.com/advanced-search (accessed on 2 February 2024)	Title Abstract Keyword—((stem cell*) AND ((bone regeneration) OR (tissue regeneration) OR (tissue engineering) OR (bone graft *)) AND (dent*) AND ((men) OR (women) OR (patient) OR (female) OR (male) OR (subjects) OR (adult)) NOT (animal models))	65
Dentistry & Oral Sciences Source—Ebscohost https://www.ebsco.com/products/research-databases/dentistry-oral-sciences-source (accessed on 2 February 2024)	TI(((stem cell*) AND ((bone regeneration) OR (tissue regeneration) OR (tissue engineering) OR (bone graft*)) AND (dent*))) AND AB(((stem cell*) AND ((bone regeneration) OR (tissue regeneration) OR (tissue engineering) OR (bone graft*)) AND (dent*))) AND ((men) OR (women) OR (patient) OR (female) OR (male) OR (subjects) OR (adult)) NOT (animal models)	2

**Table 3:** Results summary of the studies included in the systematic review.

Author (Year)	Country	Study Aim	Study Design	Stem Cell Type	Dental Surgical Procedures	Outcomes
Asahina et al. (2021) [20]	Japan	Examine the safety and efficacy of bone tissue engineering for patients with a severely atrophic alveolar bone	Cohort study	Bone marrow stem cells	Sinus lift	During treatment and follow-ups for 66 months, no side effects or health concerns were noted. Therapy was safe and effective.
Feng et al. (2021) [21]	Taiwan	Assess safety and efficacy of regeneration in the case of large bony defects	Phase I study	Small blood stem cells	Various types	All implants were implanted successfully. Elevated levels of eotaxin, fibroblast growth factor, monocyte chemoattractant protein 1, macrophage-derived chemokine, and interleukin-17A found in patients after small blood cell treatment.
Giuliani et al. (2013) [24]	Italy	Assess the stability and quality of regenerated bone and vessel network	Cohort study	Dental pulp stem cells	Bone graft	Three years after grafting in the mandible, the regenerated bone was uniformly vascularized and exhibited a compact structure, rather than a cancellous one.
Gjerde et al. (2018) [25]	Norway	Evaluate bone regeneration using marrow-derived mesenchymal stromal cells	Clinical Trial	Bone marrow-derived stromal cells	Sinus augmentation	The bone marrow cells induced significant new bone formation.
Gupta et al. (2021) [26]	India	Evaluate quality and quantity of bone formation in maxillary sinus lift and implant stability of atrophic maxilla	Case–control	Mesenchymal stem cells	Sinus augmentation	Of the 40 sinus lifts performed and 42 implants placed, all showed primary stability.
Katagiri et al. (2016) [22]	Japan	Examine safety and osteogenic potential of mesenchymal stem cells in bone	Case–control	Mesenchymal stem cells	Bone graft	Bone marrow-derived mesenchymal stem cells were used safely with less inflammation and showed great osteogenic potential.
Katagiri et al. (2017) [23]	Japan	Evaluate safety of secretome of bone marrow-derived mesenchymal stem cells for maxillary sinus lift	Case–control	Mesenchymal stem cells	Bone graft	Bone formation was clinically confirmed in all cases. The secretome of bone marrow-derived mesenchymal stem cells was used safely.

**Table 4:** Risk of bias assessment of included articles.

Author (Year)	Selection Bias ^a^	Performance Bias ^b^	Detection Bias ^c^	Attrition Bias ^d^	Reporting Bias ^e^	Other Bias ^f^	Overall Risk of Bias ^g^
Asahina et al. (2021) [20]	Low	High	Low	Low	Low	Medium	Moderate
Feng et al. (2021) [21]	Medium	High	Medium	Medium	Low	High	High
Giuliani et al. (2013) [24]	Low	Medium	Low	Medium	Medium	Medium	Moderate
Gjerde et al. (2018) [25]	Medium	High	Medium	High	Medium	Low	High
Gupta et al. (2021) [26]	Low	Medium	Low	Low	Low	Low	Low
Katagiri et al. (2016) [22]	High	High	High	Medium	High	Medium	High
Katagiri et al. (2017) [23]	High	High	Medium	Medium	Medium	Medium	High

Note: ^a^ Selection bias evaluates the risk that participants are not representative of the target population. Studies without random selection or those with small, non-diverse samples were rated as “Medium” or “High”. ^b^ Performance bias assesses the risk due to differences in care provided, often due to lack of blinding. Most studies had high performance bias due to the nature of clinical interventions. ^c^ Detection bias concerns differences in outcome assessment. Studies using objective measures (e.g., histology, radiographs) have “Low” detection bias. ^d^ Attrition bias evaluates the impact of participant drop-out on results. Studies with high dropout rates or missing follow-up data were rated as “High”. ^e^ Reporting bias assesses the risk due to selective reporting of outcomes. Studies with transparent reporting protocols were rated as “Low”. ^f^ Other bias includes additional biases such as funding bias, sample size, and center-specific issues. Ratings vary based on study specifics. ^g^ Overall risk of bias summarizes the individual domains to provide an overall evaluation.
